# Successful non-invasive imaging of the coronary artery IMT in pediatric patients with Kawasaki disease using high-resolution echocardiography

**DOI:** 10.1038/s41598-024-77345-2

**Published:** 2024-10-26

**Authors:** Stephan Gerling, Robert Dalla-Pozza, Holger Michel, André Jakob, Michael Melter, Markus Johannes Dechant

**Affiliations:** 1 Department of Pediatric Cardiology, University Children’s Hospital Regensburg (KUNO), Hospital St. Hedwig of the Order of St. John, University of Regensburg, Regensburg, Germany; 2 Department of Pediatric Cardiology and Pediatric Intensive Care, Hospital of the University of Munich, Ludwig Maximilians University Munich, 81377 Munich, Germany

**Keywords:** Kawasaki disease, Aneurysm, Atherosclerosis, Coronary arteries, Intima-media thickness, Echocardiography, Cardiology, Paediatric research

## Abstract

Kawasaki Disease (KD) is a multisystemic vasculitis of medium- and small-sized arteries. Abnormal intimal thickening may develop in the involved arterial area after regression of coronary artery aneurysm (CAA). Intimal dysfunction may induce local stenosis or arteriosclerosis in the future. In this case–control study, we investigated 29 consecutive KD patients [20 male, median current age, 7.9 years; median follow-up duration, 5.7 years] and a group of 29 healthy matched controls (CON) [19 male, median current age, 10.8 years]. They were assesed and compared for CAA, LVFS, GCS, GLS, coronary artery (CA) *Z* scores, carotid intima-media thickness (IMT) and coronary artery IMT by high-resolution transthoracic echocardiography (hrTTE). Coronary artery IMT (caIMT) was significantly higher in patients with a maximal CA *Z* score > 2.5 in acute KD than in CON: KD caIMT: 0.62 mm [IQR, 0.57–0.72 mm] vs. 0.53 mm [0.51–0.60 mm], *p* = 0.043. CAAs were found in 15 (51.7%) patients with acute KD. The maximal median LCA *Z* score in acute KD was 2.57z [IQR, 1.93—3.2z] and in follow-up −0.39z [IQR, −1.25 to −0.36z]. There was no significant difference in carotid IMT between KD patients and CON. Signs of CA intima-media thickening were detected by hrTTE in patients with a maximal CA *Z* score > 2.5 in acute KD. These data indicate that these patients may be at risk for cardiovascular sequale even in the absence of permanent CA luminal abnormalities. Therefore long-term follow-up of this group of KD patients may be required.

## Introduction

Kawasaki Disease (KD) is a multisystemic vasculitis of medium- and small-sized arteries that generally occurs in infancy and childhood. The etiology is speculated to be an exaggerated immunologic response to possible infectious or environmental triggers in genetically susceptible individuals^1^. It is considered the leading cause of acquired heart disease among children living in developed countries^2^.

The vascular inflammation process may affect arterial vessels of all body regions, but the most serious manifestation is the affection of the coronary arteries. Although intravenous immunoglobulin infusion may successfully control vascular inflammation, a certain number of patients still develop coronary artery abnormalities^3^. Therefore children with KD are at risk for serious cardiovascular sequelae, particularly coronary artery aneurysms s (CAAs), which can lead to thrombosis, coronary stenosis, myocardial infarction, and sudden death^4^.

Many autopsy studies have reported various arterial wall changes that occur in arterial segments associated with CAA, such as intimal thickening, disruption of the media, and calcification. ^5^ Only a few reports have described in vivo coronary arterial wall changes in CAAs using intravascular ultrasound (IVUS) and optical coherence tomography (OCT)^6–8^. The authors were able to demonstrate an abnormal vascular structure at the previous site of a CAA due to healing-related intimal thickening, despite the inner diameter appearing normal on echocardiography and coronary angiogram. Intimal dysfunction in the area of CAA regression may induce local stenosis or arteriosclerosis in the future^9^. However, intimal proliferation and media interruption were observed not only in areas with coronary artery aneurysms but also in regions without lesions during acute KD^8^.

Since IVUS and OCT are invasive techniques that carry periprocedural risks and expose patients to radiation, we undertook the present study to evaluate a non-invasive technique for assessing the coronary artery wall in KD patients.

We hypothesized that high resolution transthoracic echocardiography (hrTTE) will be able to detect an increase of coronary artery intima-media thickness in KD patients in order to identify patients at risk for complicated disease.

## Methods

### Study population

In this case–control study, we investigated 29 consecutive KD patients, with and without CAAs who regularly attended our KD outpatient clinic for follow-up between July 2022 and August 2023. They were matched to a group of 29 healthy nonexposed and active controls (CON) by sex and age. The prosepctive analyzed data has been collected from clinical records of patients at the children’s university hospital Regensburg (KUNO-Clinic St. Hedwig). Demographic data and records on medical, personal and family history were obtained. KD diagnoses were determined according to the current American Heart Association (AHA) guidelines^4^.

All patients received intravenous immunoglobulins (IVIG) in a dose of 2 g/kg bodyweight as a single infusion over ten hours and 40 mg/kg bodyweight acetyl salicylic acid (ASA) in four divided doses within 24 h until they were afebrile for 48 (to 72) hours. We continued to apply ASA in a lower dose (3-5 mg/kg bodyweight once per day) for 6 to 8 weeks in order to make use of its antiplatelet effect.

Patients were classified as unresponsive to IVIG if they experienced recurrent or persistent fever for at least 36 h after completing the first treatment^4^. In this case, a second dose of IVIG or additional corticosteroids (prednisolone) in a dose of 2 mg/ kg bodyweight were applied. In cases of severe refractory disease, the administration of immunomodulatory monoclonal antibody therapy (e.g. anakinra, recombinant human IL-1 receptor antagonist), cytotoxic agents, or plasma exchange may be considered as a potential treatment option in patients who have failed to respond to a second infusion of IVIG, an extended course of steroids, or a tumour necrosis factor-alpha inhibitor (infliximab) therapy.

Patients with a history of vascular or cardiovascular disease and hematological disorders were excluded from our study. To estimate the body surface area (BSA) based on the children’s height and weight, we applied the formula of Haycock^10^.

The study is in compliance with the Declaration of Helsinki and was approved by the institutional review board of the University of Regensburg (file number 14–101-0206).

### Echocardiography

Examinations were obtained using a commercially available ultrasound system (Aplio 500 CV; Toshiba Medical Systems Corporation, Otawara, Japan) with phased array transducer (PST65-BT, 4.2 MHz—9.0 MHz, Toshiba). All subjects were examined in supine or left-lateral decubitus position. The TTE evaluation was conducted by an experienced pediatric cardiologist (SG). The electrocardiogram was recorded continuously. In accordance with the American Heart Association (AHA) scientific statement on KD^4^, the proximal left main coronary artery (LMCA) was visualized using the standard parasternal short-axis view (PSAX), from the second or third intercostal space^11^. Machine controls were optimized by the operator at each examination. Optimization included altering the depth, movement of focal zone to region of the LMCA, reduction of sector size, alteration of gain control and dynamic range. Colour Doppler mapping was used to confirm visualisation of the LMCA (velocity range was set with a low Nyquist limit of 15–30 cm/s)^12^.

**Fig. 1:**
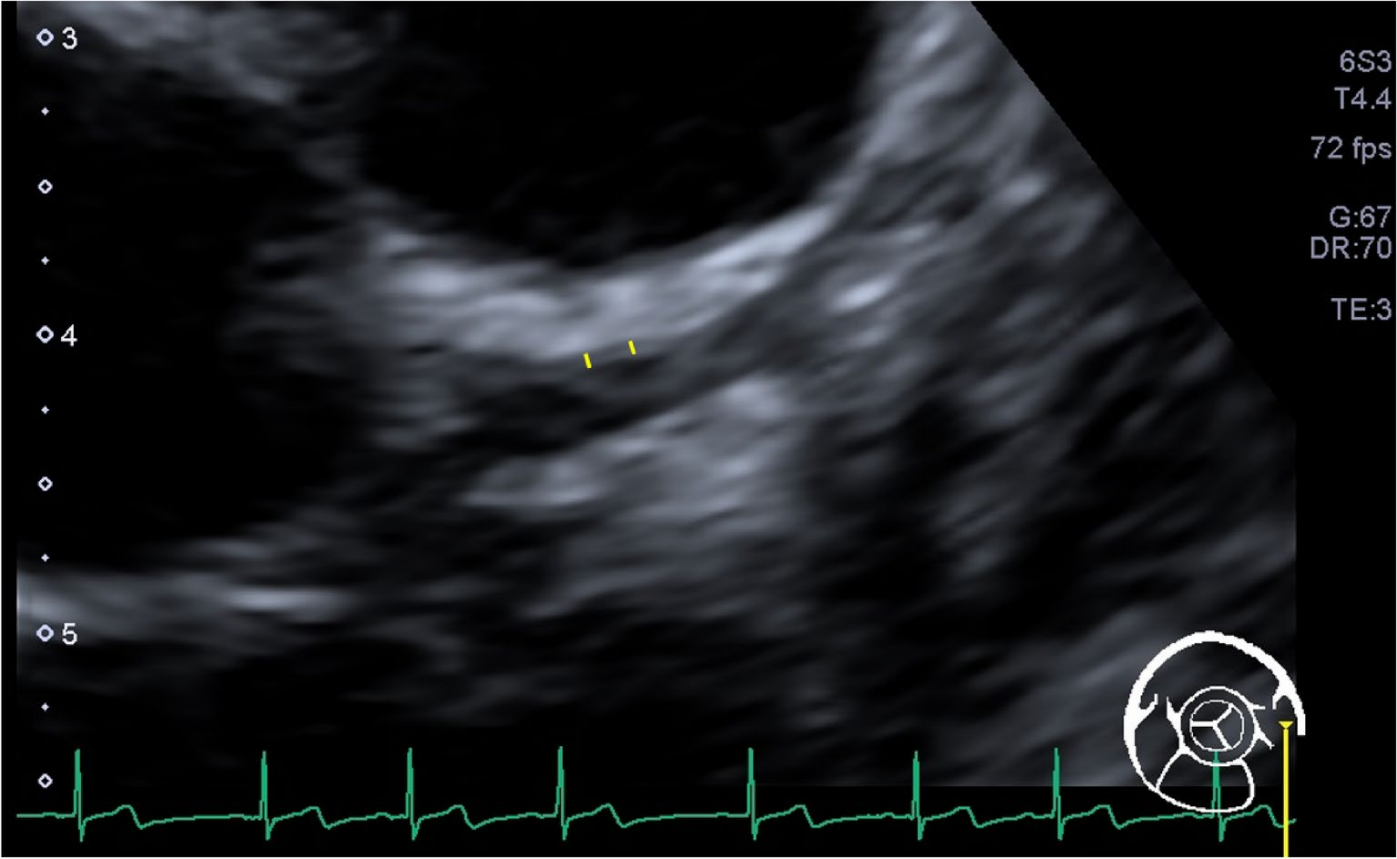
High resolution TTE, PSAX view, yellow lines indicating diastolic intima-media thickness of the left main coronary artery. Patient with a history of Kawasaki disease, eight years of age, follow-up duration 5.2 years.

In order to achieve optimal visualization of the three-layer structure of the coronary artery wall, it is essential to ensure that the ultrasound beam is oriented as perpendicular as possible. The LMCA was selected for analysis because it is identified as a horizontal tubular structure in the PSAX in the majority of cases. LMCA measurements were taken in the same area (first 5–15 mm after the origin from the aorta) for all participants. The internal diameter and coronary IMT of the LMCA were measured on the diastolic frame where the LMCA was best visualised (Fig. 1). Luminal dimensions were normalized for BSA as Z-scores according to the formula of Dallaire et al^13^.

The participants were assesed and compared for coronary artery (CA) *Z* scores, carotid intima-media thickness (cIMT) and coronary artery intima-media thickness (caIMT) by hrTTE. Carotid IMT was measured according to the guidelines from the Association for European Paediatric Cardiology (AEPC) Working Group on Cardiovascular Prevention with a 12.0 MHz linear transducer (PLT1204-BX, Toshiba)^14^. In addition, echocardiographic parameters such as left ventricular fractional shortening (LVFS), global longitudinal strain (GLS) and global circumferential strain (GCS) were recorded.

For statistical interpretation, we used averaged values from two or three single measurements. All studies were digitally stored for offline analysis, retrospective re-evaluation and follow-up examinations.

### Statistics

Continuous variables were compared using non-parametric analyses and categorical variables, using the chi-square or Fisher’s exact tests. A p-value < 0.05 was considered statistically significant. Statistical analysis was performed using IBM SPSS Statistics 25 software.

## Results

Twenty-nine KD patients [20 male, median current age, 7.9 years; median follow-up duration, 5.7 years] and 29 controls (CON) [19 male, median current age, 10.8 years] were recruitedThe median height of the KD patients was found to be 128.6 cm [IQR, 121.1 to 144.5 cm], which is lower than the median height of the controls, which was 140.8 cm [IQR, 120.4 to 158.9 cm]. There were also some differences in weight between the two groups, with a median of 26.1 kg [IQR, 22.2 to 40.8 kg] in the KD patients and a median of 37.4 kg [IQR, 21.0 to 47.2 kg] in the control group. Arterial hypertension or hypotension were not observed in the KD patients or the controls. All patients, except for one child of Asian ancestry, were of European Caucasian descent. Three patients (10.3%) received a second dose of IVIG because their fever persisted for 36 h after the initial dose. Two patients (6.9%) received corticosteroids as second-line therapy due to being IVIG refractory. Table 1 provides a summary of patient characteristics.

**Table 1:** Characteristics of Kawasaki disease patients.

KD patients (n/%)	29 (100)
Male : female	20:9
IVIG + ASA (n/%)	29 (100)
Second dose of IVIG (n/%)	3 (10.3)
Corticosteroids (n/%)	2 (6.9)
Age at the onset of KD (median)	3.25 y [IQR, 2.5 to 3.5]
Current age at hrTTE (median)	7.9 y [IQR, 6.7 to 12.3]
Follow-up duration (median)	5.7 y [IQR, 3.2 to 7.5]

*KD* kawasaki disease, *IVIG* intravenous immunoglobulins, *hrTTE* high resolution transthoracic echocardiography, *y* years, *IQR* interquartile range.

15 patients were classified as CAA + because they had a maximum CA Z score ≥ 2.5 in acute KD, and 14 patients were classified as CAA- because they had no CAA. In 28 patients, the maximum internal diameter was measured in the left main coronary artery (LMCA) or the left anterior descending artery (LAD). LCA Z scores in acute disease ranged from 0.0z to 24.03z. The median maximum LCA *Z* score in acute disease was 2.57z [IQR, 1.93 to 3.2z] and in follow-up -0.39z [IQR, -1.25 to 0.36z]. At the time of follow-up, only 3 patients still had a CAA (10.3%). This means that in 12 out of 15 patients (80%) the CAA had regressed. In this subcohort, 6 out of 12 KD patients had an elevated caIMT measurement, whereas only two participants in the overall control group had an elevated caIMT value. Coronary artery findings in KD patients are shown in Table 2.

**Table 2:** Coronary artery findings in 29 Kawasaki disease patients.

	Acute KD	Follow-up
No CAA (n/%)	14 (48.3)	26 (89.7)
CAA (n/%)	15 (51.7)	3 (10.3)
LCA *Z* max	2.57z [IQR, 1.93 to 3.2z]	− 0.39z [IQR, − 1.25 to 0.36z]
∆ *Z* max	− 2.92z [IQR, − 3.68 to − 2.39 z]	

*KD* kawasaki disease, *CAA* coronary artery aneurysm *LCA Z max* maximum median LCA *Z* score, *∆ Z max* difference between LCA *Z* max in acute KD and follow-up, *IQR* interquartile range.

The coronary IMT was significantly increased in CAA + compared to the control group, with a median of 0.62 mm [IQR, 0.57–0.72 mm] versus 0.53 mm [IQR, 0.51–0.60 mm], *p* = 0.043. CAA- patients also demonstrated a tendency towards increased coronary IMT, although statistical significance was not achieved.

There was no significant difference in carotid IMT between KD patients and the control group, despite the higher median age of the control group. Similarly, there was no significant difference in carotid IMT between KD patients without a CA aneurysm (CAA-) and patients with a CA aneurysm (CAA +) in acute disease. In CAA-, the mean carotid IMT was 0.44 mm [IQR, 0.43–0.46 mm], while in CAA + it was 0.45 mm [IQR, 0.41–0.47 mm].

The results of the carotid and coronary IMT measurements are presented in Table 3.

**Table 3:** IMT findings in Kawasaki Disease follow-up (median 5.7 years) and Controls.

	CAA-	CAA +	CON	*p*
N (%)	14 (48.3)	15 (51.7)	29 (100)	
cIMT	0.43 [IQR, 0.39–0.46]	0.45 [IQR, 0.40–0.52]	0.44 [IQR, 0.39–0.48]	N.s
caIMT	0.57 [IQR, 0.55–0.64]	0.62 [IQR, 0.57–0.72]	0.53 [IQR, 0.51–0.60]	0.043

*CAA* coronary artery aneurysm, *CAA ****-*** patients without an CAA in acute KD, *CAA* + patients with an CAA in acute KD, *CON* healthy active controls, *cIMT* carotid intima-media thickness in mm, *caIMT* left main coronary artery intima-media thickness in mm, *IQR* interquartile range, *N.s* not significant.

The *p*-value refers to the statistical comparison between CAA + and CON. A *p*-value < 0.05 was considered significant.

The study found no statistically significant difference in the functional echocardiographic parameters (LVFS, GCS, and GLS) between KD patients and controls (Table 4). Therefore, there is no evidence of myocardial dysfunction in KD follow-up patients at rest.

**Table 4:** Functional echocardiographic findings in KD patients and controls.

	KD	CON	*p*
N	29	29	
LVFS (%)	36.5 [IQR, 33.6 to 39.5]	38.1 [IQR, 34.9 to 42.5]	N.s
GCS (%)	21.1 [IQR, 19.1 to 22.4]	20.6 [IQR, 18.4 to 23.6]	N.s
GLS (%)	21.5 [IQR, 18.5 to 23.6]	21.3 [IQR, 19.2 to 24.3]	N.s

*KD* kawasaki disease patients, *CON* healthy controls, *LVFS* left ventricular fraction of shortening, *GCS* global circumferential strain, *GLS* global longitudinal strain, *IQR* interquartile range, *N.s*. not significant.

A *p*-value < 0.05 was considered significant.

## Discussion

Patients with a history of Kawasaki disease (KD) underwent high-resolution transthoracic echocardiography (hrTTE) to assess changes in the coronary arterial wall.The results were compared to those of a matched group of healthy active peers. The findings of this study indicate that KD patients who had coronary artery luminal diameters ≥ 2.5 Z score in the acute phase had a significant increased coronary artery intima-media thickness in medium term follow-up. Patients without coronary artery aneurysms (CAAs) in the acute phase also demonstrated a tendency towards elevated coronary IMT, although statistical significance was not achieved.

Many autopsy studies have reported arterial wall changes in arterial segments associated with CAA in acute KD, such as intimal thickening, media disruption, and calcification. These structural findings are associated with functional abnormalities and could precede clinical events, reflecting chronic pathological vascular processes in patients with KD^5^.

In a recent systematic review, Lee JJY et al. reported that major adverse cardiac events (MACE) occurred primarily in KD patients with coronary aneurysms. A MACE-free survival varied from 36 to 96% at 30 years after acute KD^15^. The authors also reported evidence of an increased risk of early atherosclerosis in patients with a history of KD complicated by CAA^16^.

Iemura et al. studied KD patients with various degrees of coronary artery involvement who had all progressed to a normal luminal dimension on angiography. Abnormal intimal thickening developed in the involved arterial area after CAA regression, although the inner diameter appears normal on echocardiography and coronary angiography. However, the function of endothelial cells in these remodeled vessels was abnormal.The authors noted that segments that previously had large aneurysms exhibited paradoxical vasoconstriction in response to acetylcholine and reduced vasodilation in response to nitroglycerin^17^. Takahashi et al. concluded that intimal dysfunction in the area of CAA regression may induce local stenosis or arteriosclerosis in the future^18^. Apart from endothelial dysfunction, patients with a history of KD are also prone to develop, arterial stiffening^19^. and a proatherogenic lipid profile^20^. KD patients exhibited persistently low levels of high-density lipoprotein cholesterol^21^ and patients with a history of KD complicated by CAA showed atherogenic lipid profiles^22^.

Although KD is recognized as an acute inflammatory vascular disease, several reports^22–24^ have suggested ongoing inflammation. Patients with a history of KD complicated by CAA showed increased high-sensitivity C-reactive protein and/or serum amyloid A. In addition, adult patients who had giant CAA caused by KD showed significantly higher proinflammatory plasma calprotectin than those who had smaller CAA or healthy volunteers^25^. Suda et al. recently conducted a study on patients with a history of KD using X-ray computed tomography and 18F-fluorodeoxyglucose positron emission tomography. Their findings suggest that vascular inflammation may persist many years after KD, particularly in patients with severe inflammation expressed as giant CAA in the acute phase^26^.

Conventional non-invasive imaging methods, such as magnetic resonance imaging (MRI) or computed tomographic (CT) angiography, do not provide optimal resolution of wall thickness^27^ and may require complex and expensive technologies, CT potentially exposing patients to radiation risk^28^. Invasive methods such as intravascular ultrasound (IVUS), optical coherence tomography (OCT) and optical frequency domain imaging (OFDI) can provide high-resolution images and precise information about the structure of the coronary artery wall. IVUS has been used in patients with a history of KD to demonstrate both symmetrical and asymmetrical wall thickening in aneurysms, particularly in those aneurysmal segments that have progressed towards normal luminal dimensions^17,29^. In another study using IVUS, Tsuda et al. also found that intima–media thickening > 400 μm frequently developed in coronary branches that dilated to > 4.0 mm diameter within 100 days of KD onset^6^.

Furthermore, Dionne and colleagues demonstrated significant alterations in the arterial wall structure of KD patients through the use of optical coherence tomography (OCT) imaging^6^. Intimal hyperplasia was the most frequent finding in their study cohort and was observed in 83.3% of their patients. The thickness of the intima was significantly different between diseased and normal coronary artery segments (390.8 ± 166.0 μm versus 61.7 ± 17.0 μm).

Since IVUS, OCT and OFDI are invasive techniques that carry periprocedural risks and expose patients to radiation, it makes sense in the interest of the patient to have a non-invasive imaging technique that has the ability to detect KD patients at risk for atherosclerosis.

Labombarda et al. evaluated wall thickness of the LMCA examined by transthoracic echocardiography (TTE) in coronary patients and compared these with findings obtained by optical frequency domain imaging (OFDI). A significant positive correlation between TTE and OFDI for the anterior wall thickness of the LMCA was detected. The authors concluded that transthoracic echocardiography may offer a non-invasive alternative to imaging modalities for measuring coronary wall thickness^30^.

Recent research has focused on carotid IMT to determine whether patients with a history of KD may be at risk of premature atherosclerosis. Some studies have shown that the mean carotid IMT in KD patients is significantly higher than in controls, while other studies have not shown similar results^14,31,32^. Using high-resolution ultrasound probes in TTE, Ruscica et al. showed a significant correlation between left main coronary artery (LMCA) wall thickness and carotid IMT in patients referred to a Lipid Clinic^33^.

Our study found no significant difference in carotid IMT between KD patients and healthy, active controls who were not exposed to KD, although the median age of the control group was higher than that of the patients.

Our findings support the hypothesis that an increase in the IMT of the anterior wall of the left coronary artery, as determined by TTE, could predict coronary atherosclerosis and clinical events in patients with a history of KD complicated by CAA. Currently, there are no standard values for coronary artery wall diameter or coronary IMT in the paediatric population. Further research should be conducted to evaluate coronary IMT in paediatric populations with a higher risk of developing atherosclerosis using hrTTE.

## Limitations

The present study has limitations and should be viewed as a pilot study. The study had a limited number of participants, and most of the patients were Caucasians from Continental Europe. It should be noted that the follow-up period is only medium-term. As a result, the study’s findings can only provide an estimate of the long-term effects. As blood lipids were not measured, it cannot be ruled out that an increase in coronary IMT could also be caused by elevated levels of blood lipids. However, no increased values were found for carotid IMT in the same participants. It would have been desirable to use a technique such as intravascular ultrasound or optical coherence tomography in the participants to assess the accuracy of the sonographic measurement of the coronary IMT.

## Conclusions

Signs of coronary artery intima-media thickening were detected by high-resolution TTE in patients with a maximal coronary artery *Z* score ≥ 2.5 in acute Kawasaki disease. These data indicate that these patients may be at risk for cardiovascular sequale even in the absence of permanent coronary artery luminal abnormalities. Therefore long-term follow-up of this group of Kawasaki disease patients may be required. High-resolution TTE may be a promising non-invasive tool for assessing the vessel wall in patients at risk of developing coronary atherosclerosis.

## Data Availability

The datasets used and/or analysed during the current study available from the corresponding author on reasonable request. corresponding author on reasonable request.

## Abbreviations

BSA: Body surface area
CA: Coronary artery
CAA: Patients without a CA aneurysm in acute KD
CAA +: Patients with a CA aneurysm in acute KD
CT: Computer tomography
GCS: Global circumferential strain
GLS: Global longitudinal strain
hrTTE: High resolution transthoracic echocardiography
IQR: Interquartile range
IMT: Intima-media thickness
IVUS: Intravascular ultrasound
KD: Kawasaki disease
LMCA: Left main coronary artery
LAD: Left anterior descending artery
LCX: Left circumflex coronary artery
LV: Left ventricle
LVFS: Left ventricular fraction of shortening
MACE: Major adverse cardiac events
MRI: Magnetic resonance imaging
OCT: Optical coherence tomography
PSAX: Parasternal short axis view
PLAX: Parasternal long axis view
RMCA: Right main coronary artery
SD: Standard deviation
TTE: Two-dimensional transthoracic echocardiography
